# Antisense Peptide Technology for Diagnostic Tests and Bioengineering Research

**DOI:** 10.3390/ijms22179106

**Published:** 2021-08-24

**Authors:** Nikola Štambuk, Paško Konjevoda, Josip Pavan

**Affiliations:** 1 Center for Nuclear Magnetic Resonance, Ruđer Bošković Institute, Bijenička cesta 54, HR-10000 Zagreb, Croatia; 2 Laboratory for Epigenomics, Division of Molecular Medicine, Ruđer Bošković Institute, Bijenička cesta 54, HR-10000 Zagreb, Croatia; 3 Department of Ophthalmology, University Hospital Dubrava, Avenija Gojka Šuška 6, HR-10000 Zagreb, Croatia

**Keywords:** antisense, complementary, peptide, binding, genetic code, technology, bioengineering, SARS-CoV-2

## Abstract

Antisense peptide technology (APT) is based on a useful heuristic algorithm for rational peptide design. It was deduced from empirical observations that peptides consisting of complementary (sense and antisense) amino acids interact with higher probability and affinity than the randomly selected ones. This phenomenon is closely related to the structure of the standard genetic code table, and at the same time, is unrelated to the direction of its codon sequence translation. The concept of *complementary peptide interaction* is discussed, and its possible applications to diagnostic tests and bioengineering research are summarized. Problems and difficulties that may arise using APT are discussed, and possible solutions are proposed. The methodology was tested on the example of SARS-CoV-2. It is shown that the CABS-dock server accurately predicts the binding of antisense peptides to the SARS-CoV-2 receptor binding domain without requiring predefinition of the binding site. It is concluded that the benefits of APT outweigh the costs of random peptide screening and could lead to considerable savings in time and resources, especially if combined with other computational and immunochemical methods.

## 1. Introduction

**Figure 1:**
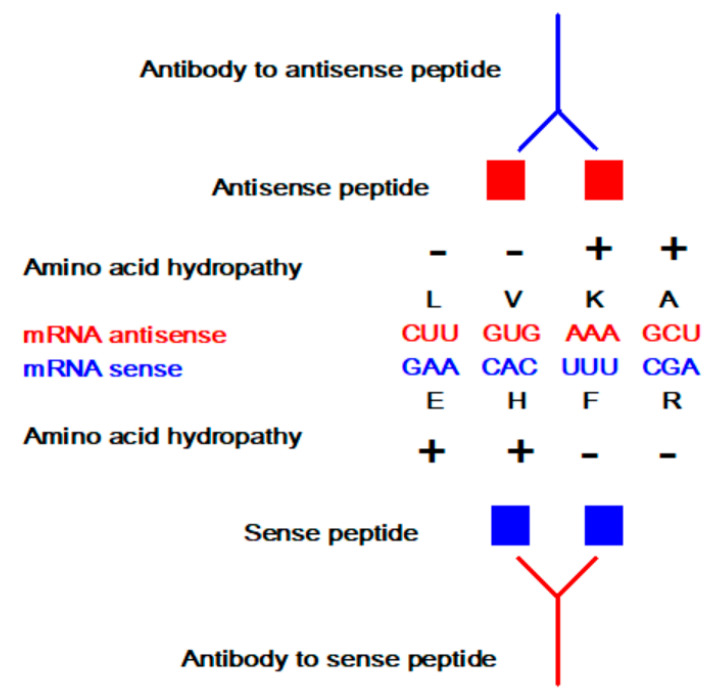
Modeling of sense-antisense peptide binding and related epitope-paratope interactions.

The concept of sense and antisense (i.e., complementary) peptide interaction was developed in the early 1980s by Root-Bernstein, Biro, Blalock, Mekler, Siemion, and others [1,2,3,4,5,6,7,8,9,10,11,12,13,14,15,16,17,18,19,20]. First, it was theoretically assumed and later empirically observed that peptides consisting of amino acids specified by sense and antisense sequences interact with higher probability and affinity than randomly selected peptides (Table 1 and Table 2, Figure 1). This approach was successfully applied to the investigations of more than 50 ligand–acceptor (receptor) systems, including the immune response to viral subunits and related manipulations with an epitope and paratope design [1,2,3,4,5,6,7,8,9,10,11,12,13,14,15,16,17,18,19,20,21,22].

Sense peptides are essential and specific parts of viral and other proteins that elicit normal and pathologic immune responses [6,7,8,9,14,19,20,21]. Using antisense peptide technology, they could be utilized to derive targeted tests for different antibody (Ab), hormone, growth factor, or cell subpopulations [4,5,6,7,8,9,13,14,17,18,19,20,21,22,23]. The potential of antisense peptides is twofold: 1. as future diagnostic tests targeting protein epitopes or paratopes of interest, or 2. as future therapeutic agents that target specific parts of antigens to selectively modify host immune response (e.g., an antisense peptide may disrupt or modify different factors like virulence, replication or host defense) [6,7,8,9,13,14,18,19,20,21]. Consequently, sense-antisense peptide interactions may serve as a useful starting point for: 1. the development of biochemical assays for the evaluation of the immune response, and 2. modeling and design of new peptide binders for specific proteins and their receptors.

## 2. Antisense Peptide Technology (APT)

The antisense peptide binding preference for the complementary sense sequence provides the opportunity to build a technology platform for the development and implementation of new immunochemical procedures and assays which use antisense peptides instead of the primary and/or secondary antibodies. APT is based on a heuristic algorithm for rational peptide design of the interacting ligand-receptor (acceptor) sequences specified by the complementary codons (Table 1 and Table 2, Figure 1) [1,2,3,4,5,6,7,8,9,10,11,12,13,14,15,16,17,18,19,20,21,22,23,24]. Heuristic methods reduce solution space by focusing on results based on the reduced set of criteria—in this case, complementarity rules defined by the standard genetic code (SGC) table [19,24].

Four main problems of sense and antisense peptide applications in immunochemistry are comparable to the reasons for the lack of success of the synthetic peptide vaccines [25], while the fifth topic addressed is related to the application of antisense/complementary sequences in bioengineering [18,19,20,24]:

1. reliance on continuous epitopes,
2. overconfidence in ligand specificity,
3. amino acid bias in characterizing ligand-acceptor (receptor) interactions,
4. difficulties in the estimation of structure-function relationships between specific ligand–acceptor (receptor) pairs,
5. amino acid coding, complementarity, and frameshifts.

Each of these specific problems is worth addressing.

### 2.1. Reliance on Continuous Epitopes

Epitopes and paratopes are not structural features of molecules [25,26]. They are entities characterized by a recognizable identity and defined by mutual complementarity [25,26]. Edmundson et al. [25,26,27] proposed the contact model of “flexible keys and adjustable locks” for epitope and paratope interaction. An epitope may be characterized as continuous and discrete [25,26]. Modeling of continuous epitopes by means of APT is often used for the sequences between 5 and 15 amino acids. The application of APT to discontinuous epitopes, between 10 and ≈20 amino acids, is more complicated, and similarly to Abs, often requires complex procedures that involve precise definition in structural terms, i.e., X-ray crystallography [7,20,25]. Discontinuous epitopes consist of amino acid side chains of two to five separate protein fragments that are brought together by the folding of the peptide chains—which act as a scaffold [25].

Recent computational docking methods for protein and peptide interactions, and progress in peptide library-use concerning synthetic and/or structurally modified peptides, enable comparative studies and engineering of both continuous and discontinuous peptides and selection of potential motifs/lead compounds [20,28,29,30,31]. Novel protein-peptide docking procedures are based on different aspects of interaction studies, including inhibitor screening, model prediction, experimental data interpretation, specificity of prediction, and design of interfering peptides [28].

### 2.2. Overconfidence in the Ligand Specificity

**Figure 2:**
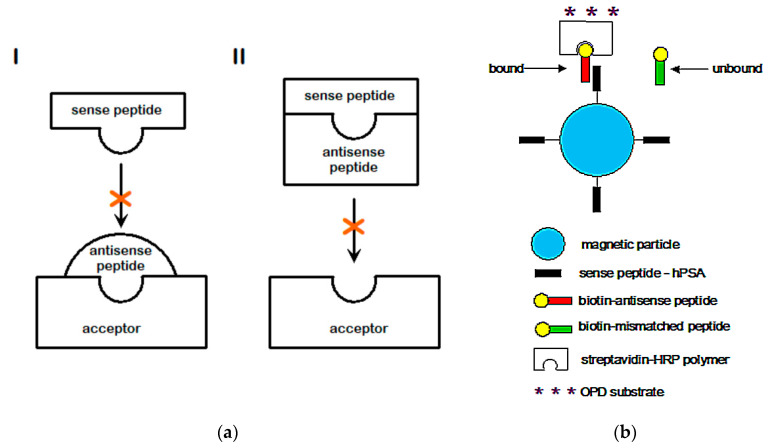
(**a**) Possible interactions of the complementary peptides: (I) acceptor-antisense binding, and (II) sense-antisense binding. (**b**) Schematic representation of peptide-based magnetic particle enzyme immunoassay (MPEIA) according to Štambuk et al. [20].

Selective targeting of peptide motifs (epitopes) could be achieved via APT (Figure 2a), with certain advantages, disadvantages, and differences with respect to the antibodies. When an antisense peptide is used, its small size—in comparison with the antibody—enables depth of tissue penetration [20]. The binding affinity measurements for antisense peptides are often in the micromolar *K*_d_ range, while the values for the antibodies are in the nano- to micromolar range, and maximum care must be applied to the selection of peptides with optimal affinity, i.e., the lowest possible *K*_d_ [19,20].

Different methods have been used to evaluate sense-antisense peptide interactions, ranging from microtiter plate assay methods (immunoassays) to high-performance affinity chromatography, and other related techniques [2,3,4,5,6,12,13,14,15,18,19,20].

Standard biochemical methods for this type of analysis include enzyme-linked immunosorbent assay (ELISA), magnetic particle enzyme immunoassay (MPEIA, Figure 2b), and microscale thermophoresis. Meanwhile, the use of other methods, usually depending on the experimental design, includes tryptophan fluorescence spectroscopy, biosensor-based surface plasmon resonance, resonant mirror (RM) biosensor assays, electrospray ionization mass spectrometry, and NMR spectroscopy [2,3,4,5,6,12,13,14,15,18,19,20,32]. Although the results of binding affinity measurements may vary from method to method, recent comparative measurements involving tryptophan fluorescence spectroscopy, microscale thermophoresis (MST), and MPEIA showed consistent results for these simple, quick, and inexpensive methods that could be used for high-throughput screening [18,19,20]. However, binding affinity and biological activity are not synonymous because high affinity is not necessarily accompanied by high activity [32,33].

### 2.3. Amino Acid Bias in Characterizing Ligand-Acceptor (Receptor) Interactions

The huge difference in the number of possible antisense peptides available for sequence selection and possible database screening depends on the direction of the mRNA translation. The standard genetic code table specifies the translation of antisense (or complementary) peptides in two directions. Table 2 shows that 27 antisense amino acid pairs are derived by the 3′ → 5′ translation direction and significantly more (52 pairs) are obtained using the 5′ → 3′ direction algorithm [16,17,18,19,20]. The latter result is due to the fact that 5′ → 3′ antisense translation of the genetic code is based on 16 groups of codons, while 3′ → 5′ antisense translation depends on only four codon groups [19]. According to Siemion et al. [13,19], there are three main hypotheses concerning the interaction of sense-antisense peptides based on complementary coding principles.

The Mekler-Blalock antisense hypothesis is based on the hydropathic complementarity principle of sense and antisense peptide interactions, named Molecular Recognition Theory (MRT), which is independent of the direction of triplet reading, since the central (second) base of the coding triplet specifies the hydropathy of the amino acid [7,8,9,13,19].

According to Root-Bernstein, the antisense approach in the 3′ → 5′ direction applies to peptides of <20 amino acids that may lack specific secondary and tertiary structure [3,4,5,6,18,19,20]. Such design leads to significantly fewer antisense peptides and represents a plausible solution for the screening of bioactive ligands [3,4,5,6,18,19,20].

The Siemion hypothesis of sense-antisense peptide interaction is based on the periodicity of the genetic code, i.e., the Siemion one-step mutation ring of the code, and the resulting sense-antisense amino acid pairs are in most cases similar to the 3′ → 5′ translation direction [13,19].

The clustering of amino acid pairs, according to interaction preference, is defined by the complementary U ↔ A and C ↔ G bases of the second codon base. The second codon base, according to Woese, specifies the physicochemical properties of the amino acids [24,34]. Therefore, it is not surprising that diverse amino acid properties—like hydrophobicity, hydrophilicity, lipophilicity, and molecular descriptors of contact potential (Miyazawa-Jernigan), hydrophobic moment, and intrinsic disorder—follow the identical sense and antisense complementarity clustering scheme that is associated with molecular interaction at the peptide level (≥4 aa) [7,8,9,10,11,12,13,14,15,16,17,18,19,20,21,22,23,24]. In a recent article, Štambuk et al. [19] emphasized that “the natural genetic coding algorithm for sense and antisense peptide interactions combines elements of amino acid physico-chemical properties, stereochemical interactions, and bidirectional transcription”. The relationship of the genetic code and amino acid polarity with respect to protein structure and temperature conditions are discussed in reference [24] and the related Data in Brief article.

### 2.4. Difficulties in the Estimation of Structure-Function Relationships between Specific Ligand-Acceptor (Receptor) Pairs

The effects of an antisense ligand on its sense receptor (acceptor) may arise from the biological modulation and/or neutralization of the sense peptide effects by means of [6,7,17,35]:

- peptides binding into molecular complexes (leaving none or low levels of sense peptide to elicit its own biological effects),
- total or partial antagonization of the sense peptide receptor by means of its complexation with an antisense ligand,
- combination of the first two factors,
- other biological or biochemical effects of an antisense peptide that cannot be explained by the involvement of a sense peptide and its receptors (e.g., generation of bioactive antibodies to peptides and/or their complexes, cellular receptor, and growth factor modulation).

Each of those points should be carefully analyzed in the context of receptor binding and the biological effects observed under specific experimental designs. For example, in proliferative and biochemical studies involving cellular receptors and serum/plasma proteins, experimental results may be modified by the anticoagulant used—heparin, citrate, or EDTA [36,37].

Amino acid isomerization may also be an important factor in modifying protein or peptide structure, interaction, and receptor binding properties [16,19,33,38,39,40].

### 2.5. Amino Acid Coding, Complementarity, and Frameshifts

Amino acid coding with respect to complementary protein constructs, mutation, and frameshifts have been studied by many authors, including Arques and Michel [41,42], Bartonek et al. [43], McGuire and Holmes [21], Štambuk [44,45], Wichmann et al. [46,47], and Youvan et al. [48,49,50].

A recent article by Bartonek et al. [43] showed that a frameshifting mechanism could be an effective evolutionary strategy for generating novel proteins with mostly unchanged physicochemical properties. Nevertheless, an important aspect of frameshift coding related to antisense/complementary sequences needs to be addressed. In 1996, Arques and Michel [41,42] identified a complementary circular code of trinucleotides (X) which on average has the highest occurrence in the reading frame (X_0_) compared to the two shifted frames (X_1_ and X_2_).

This code was found in the protein coding genes of bacteria, archaea, eukaryotes, plasmids, and viruses [42,51]. It enables the reading frames to be retrieved in genes without start codons and with a window length of ≥13 nucleotides [41,42]. The frame X_0_ consists of 12 amino acids (A, N, D, Q, E, G, I, L, F, T, Y, V), while frames X_1_ (A, R, C, I, L, K, M, P, S, T, V) and X_2_ (A, R, C, Q, G, H, L, P, S, W, Y) have 11 amino acids each [41,42,51]. With respect to the antisense codon and amino acid translation in the 5′ → 3′ direction, the X_0_ frame of the circular code is self-complementary, and X_1_ and X_2_ frames are mutually complementary [41,42]. In 1999, Štambuk showed that the combinatorial necklace model enables the use of coding theory arithmetic in the analyses of the genetic code and circular code antisense translations [24,44,45,52].

Two seemingly opposite biological coding rules are characteristic for the interpretation of the SGC frameshifts and related mathematics—including complementary transformations within frames. They both deal with the mechanisms of translation error-control and flexibility and could have an important impact on SGC repertoire manipulations.

The first coding rule is that X_0_, X_1,_ and X_2_ frames of the circular code distinguish three possible reading frames of the protein-coding sequence since hidden stop codons in X_1_ and X_2_ prevent off-X_0_-frame protein translation—this procedure is often named ambush hypothesis [53,54], and it is thought to ensure accurate translation.

Paradoxically, the second coding rule—related to SGC flexibility—is that stop codon readthrough may be promoted by the nucleotide environment, with glutamine (Q), tyrosine (Y), and lysine (K) inserted at UAA and UAG stop codons, whereas tryptophan (W), cysteine (C), and arginine (R) could be inserted at a UGA stop codon [55,56].

Considering bioengineering modeling, a reduced number of amino acids in frames X_0_, X_1_, and X_2_ match the criteria for the use of simplified amino acid alphabets for engineering purposes and related sample space reductions [57]. Consequently, we measured the relationships of the main amino acid (aa) properties addressed by Bartonek et al. [43] in the frames X_0_, X_1_, and X_2_ of the complementary circular code [41,42]. The factors of amino acid polarity, secondary structure, molecular volume, diversity, and electrostatic charge by Atchley et al. [58] were correlated to scales of nucleobase/amino acid interaction preferences for guanine (GUA), purines (PUR), and pyrimidines (PYR) [43,59].

A significant rise in the correlation of amino acid polarity to preference scales for guanine GUA, PUR, and PYR was observed in frame X_0_ (Table 3). In frame X_1_ (shifts +1 and −2), we found a strong correlation between codon and amino acid diversity factor and GUA, PUR, and PYR scales (Table 3). This observation is not surprising, since Atchley et al. [58] reported that diversity factor exhibits a highly significant correlation to amino acid physiochemical attributes and substitution matrices, and the X_1_ frame is specified by the second codon base, which is associated with the majority of such information [24,34,60].

However, in frame X_2_ (shifts +2 and −1), correlations between physiochemical factors and nucleobase preference scales were not significant. This observation is in agreement with recent findings that, contrary to X_1_, the frame X_2_ of the complementary circular code is less optimized than the SGC to reduce the effects of +2 and −1 frameshifts, in particular with respect to the physicochemical properties of amino acids [51].

A rise in correlation among amino acid factors and nucleobase preference scales in frames X_0_ and X_1_ of the circular codes may reflect the importance of the first two bases for the variables encoding scheme [24,34,60], and points to a possible application of GUA, PUR, and PYR scales [43,59] to different genetic code analyses. In our opinion, comparative investigations of complementary circular code and SGC—concerning frameshifts, error-correction, evolution, and biological engineering—seem to be justified.

As emphasized by Choi et al. [61], “ribosome is intrinsically susceptible to frameshift before its translocation and this transient state is prolonged by the presence of a precisely positioned downstream mRNA structure”. Additionally, according to Rozov et al. [62], ribosome also “prohibits the G-U wobble geometry at the first position of the codon–anticodon helix”. Therefore, it is not surprising that programmed ribosomal frameshifting enables reverse-genetics approaches and the construction of modified viruses with engineered deletions and/or foreign inserts [63].

Such engineering procedures could be used: 1. for artificial control of gene expression at the translation level, and 2. to generate differentiable marker vaccines and modified live virus vaccines [61,63]. More details on the challenges and perspectives of reverse vaccinology (RV) approaches may be found in Van Regenmortel [64] and Moxon, Reche, and Rappuoli [65].

## 3. An Example of SARS-CoV-2

One potential application of antisense peptide technology is related to the SARS-CoV-2 receptor-binding domain (RBD). The SARS-CoV-2 entry into human cells is characterized by the binding between viral spike (S) protein RBD and its receptor protein, i.e., angiotensin-converting enzyme II (ACE2) [66,67,68,69]. Consequently, a promising area of SARS-CoV-2 research is the disruption of SARS-CoV-2 binding to ACE2 by means of designer drugs and/or peptides [70,71,72]. Glasgow et al. [73] applied a stepwise engineering approach and generated enzymatically inactivated ACE2 variants, i.e., receptor traps, to potentially block RBD-ACE2 binding and entry into host cells to prevent infection. They used a position distant from the enzyme active site but situated at (or near) the place of enzyme contact with the virus-binding domain [73]. 

A similar approach may be taken by using an antisense peptide design. The ACE system was one of the classic targets for the investigation of sense-antisense peptide interactions decades before the COVID-19 crisis [3,6,15,16,17,74], and Huang et al. [75] used APT translation in the 5′ → 3′ direction to screen a potential inhibitor for SARS-CoV.

**Figure 3:**
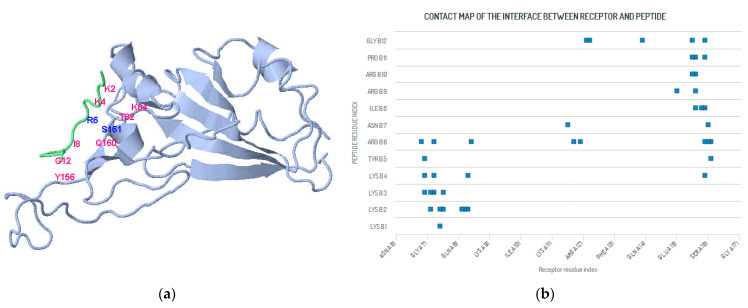
(**a**) Docking of peptide KKKKYRNIRRPG to RBD of S1 SARS-CoV-2 protein by means of CABS-dock server (Table S1: model 1) [28,75]. Pairs of peptide/receptor residuals shown in magenta or blue (3′ → 5′ antisense); (**b**) Contact map of the interface between peptide and receptor residues in a complex closer than 4.5 Å (Table A2 and Table S1: model 1).

Huang et al. [75] identified a dodecapeptide KKKKYRNIRRPG with high-binding affinity and specificity to the S protein of SARS-CoV and concluded that it could be used as a lead compound for further inhibitor studies. We additionally analyzed the docking of KKKKYRNIRRPG to the S1 protein of SARS-CoV-2 by means of a CABS-dock server (Figure 3, Table A1, Table A2 and Table S1). CABS-dock is a global docking procedure with explicit fully flexible docking simulation and clustering-based scoring, in which receptor flexibility is limited by default to small backbone fluctuations (but can be increased to include selected receptor fragments) [28]. We observed docking of the peptide ligands to the 190 residue fragments (aa 334–523) of S1 RBD known to be responsible for virus binding (Table A1) [66,68,69]. The results presented in Figure 3 and Table A2 indicate that KKKKYRNIRRPG binds RBD S1 of SARS-CoV-2 at positions Y156 (aa 489), L159 (aa 492), Q160 (aa 493), and S161 (aa 494)—that belong to the region relevant for receptor binding [66,68,69,73]. This confirms the validity of the APT approach and applicability of the methods of Huang et al. [75] for SARS-CoV-2 molecular design.

**Figure 4:**
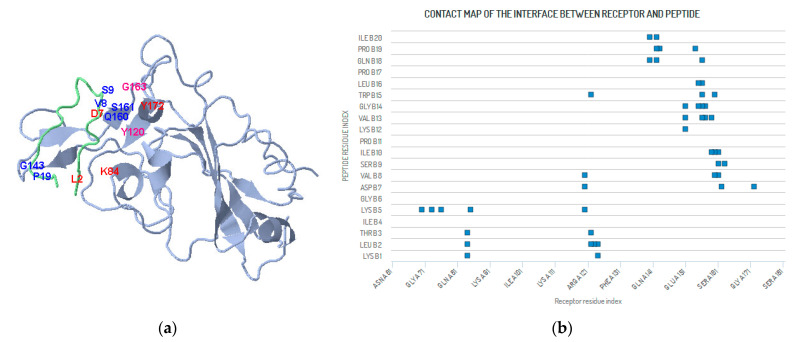
(**a**) Docking of antisense peptide KLTIKGDVSIPKVGWLPQPIV to RBD of S1 SARS-CoV-2 by means of a CABS-dock server (Table S2: model 1) [28]. Pairs of peptide/receptor residuals: in 3′ → 5′ direction marked blue, in 5′ → 3′ direction marked red, otherwise magenta; (**b**) Contact map of the interface between antisense peptide and its receptor residues in a complex closer than 4.5 Å (Table A3 and Table S2: model 1).

**Figure A1:**
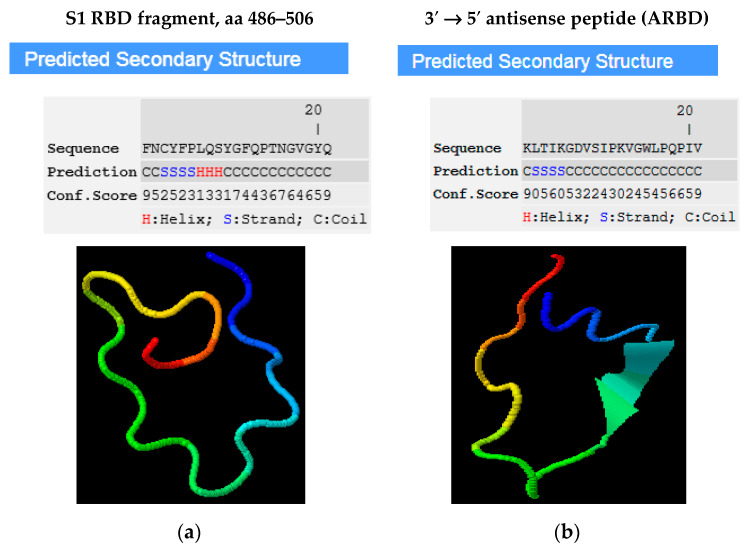
(**a**) Receptor binding domain fragment aa 486–506 of S1 SARS-CoV-2 protein (PDB file: Table S5), and its antisense peptide translated in 3′ → 5′ direction (PDB file: Table S6) (**b**). Peptide structure prediction and modeling was done using I-TASSER (Iterative Threading ASSEmbly Refinement) [80].

The validity of the 3′ → 5′ antisense approach was tested using the same methodology. We designed a 3′ → 5′ antisense paratope motif KLTIKGDVSIPKVGWLPQPIV complementary to the S1 RBD sequence of SARS-CoV-2 FNCYFPLQSYGFQPTNGVGYQ at sequence positions spanning from F153 to Q173 of the RBD S1, i.e., its aa 486–506 region (Figure A1). The docking was done using a CABS-dock server (Figure 4, Table A3). The results, presented in Figure 4 and Table A3, accurately predict the binding of 3′ → 5′ antisense peptide paratope KLTIKGDVSIPKVGWLPQPIV to its sense S1 RBD receptor epitope FNCYFPLQSYGFQPTNGVGYQ. Important contact positions are Y156 (aa 489), L159 (aa 492), Q160 (aa 493), S161 (aa 494), G163 (aa 496), and Y172 (aa 505), which are within the region that is relevant for receptor binding [66,68,69,73]—which points to the applicability of APT for the modeling of sense-antisense peptide interactions in SARS-CoV-2 research.

APT, as a heuristic method, may also contribute to the rationalization of peptide library screening [18,19,20,24,32]. The heuristic approach to problem-solving is a practical method that employs an empirical procedure that may not always be optimal or perfect but is sufficient for finding a satisfactory approximate solution [76,77,78]. In computer science, bioinformatics, and mathematical optimization, this technique is often used as a shortcut for problem-solving in situations when classic methods are too slow or when they fail to provide the exact solutions [76,77,78]. The genetic coding algorithm for complementary (sense and antisense) peptide interactions [19] belongs to this class of so-called “heuristic algorithms”. Their basic property is a trade-off between speed and completeness [78]. They are often used to solve NP-complete problems, i.e., decision problems without a known way to find a solution that is quick and efficient. The solution obtained via a heuristic algorithm can be used directly, or it may represent a good point for the application of exact optimization algorithms. For example, to find all possible random amino acid binders for a peptide 5 aa in length, we need to examine 3.2 × 10^6^ solutions (20^5^). On the other hand, using APT, the range of possible solutions is 1–243 for the 3′ → 5′ and 1–1024 for the 5′ → 3′ translation, with ≈32 (≈2^5^) as the average number of solutions. This represents a reduction of solution space by a factor of 1 × 10^5^. For longer sequences, this difference is even more pronounced. A heuristic solution could be easily checked experimentally, and APT is a typical example of that since it has been confirmed to be valid for >50 ligand-acceptor systems [4,5,6,7,8,9,12,13,14,15,16,17,18,19,20,21,22,23]. Computational models have their cost (approximately 10 USD/model) [79], which means that the reduction of possible solution space has a significant influence on the search outcome.

**Figure 5:**
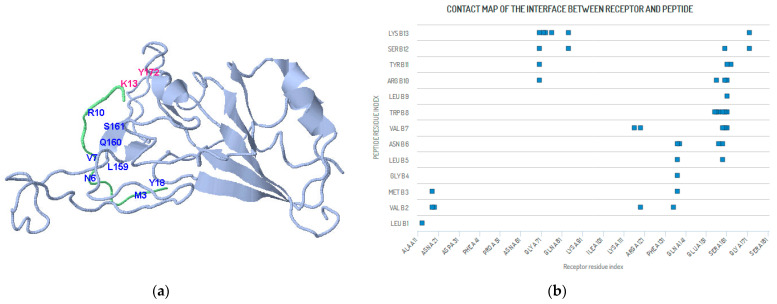
(**a**) Docking of peptide LVMGLNVWLRYSK to RBD of S1 SARS-CoV-2 protein by means of CABS-dock server (Table S3: model 1) [28,29]. Pairs of peptide/receptor residuals: in 3′ → 5′ direction marked blue, otherwise magenta; (**b**) Contact map of the interface between peptide and its receptor residues in a complex closer than 4.5 Å (Table A4 and Table S3: model 1).

For example, Pomplun et al. [29,30] identified a consensus peptide sequence LVMGLNVWLRYSK that binds RBD S1 of SARS-CoV-2 using random library screening with 800 million synthetic peptides. The results of the CABS-dock procedure, presented in Figure 5 and Table A4, show that LVMGLNVWLRYSK is likely to bind the RBD S1 fragment at positions identical to the ones detected using the APT approach: Y156 (aa 489), L159 (aa 492), Q160 (aa 493), S161 (aa 494), G163 (aa 496), and Y172 (aa 505).

## 4. Methods and Results (SARS-CoV-2 Peptide Modeling)

### 4.1. Peptide Modeling and Peptide–Protein Docking

The fragment of S1 RBD sequence aa 334–523 (190 aa, Table A1)—reported to be responsible for SARS-CoV-2 binding [66,68,69]—was used for protein modeling, design, and docking [28]. Input PDB file, Table S4, was obtained using I-TASSER (Iterative Threading ASSEmbly Refinement) [80].

**Figure 6:**
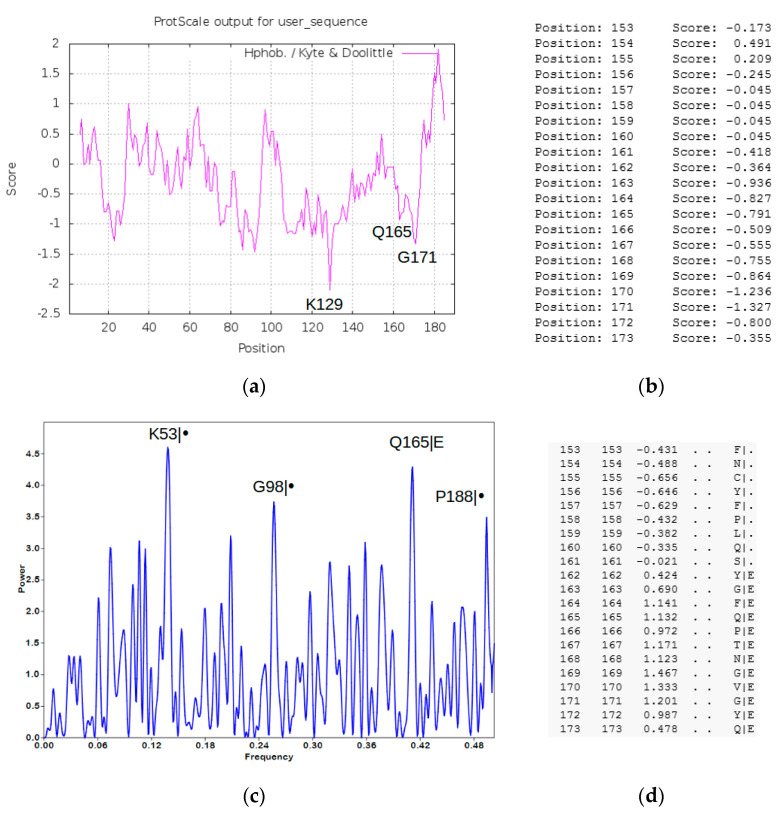
S1 epitopes of SARS-CoV-2 RBD sequence fragment (aa 334–523) were determined with Expasy—ProtScale tool using the hydrophobicity scale of Kyte & Doolittle and a sliding block of 11 amino acids [81,82] (**a**). Hydropathy scores for the epitope positions at aa 486–506 (153–173) (**b**). Informational Spectrum Method analysis based on the electron-ion interaction pseudopotential was used to determine the bioactive region (Q165) of the SARS-CoV-2 RBD sequence fragment (aa 334–523) [18,83,84] (**c**). Prediction of B cell epitopes of the SARS-CoV-2 RBD sequence fragment with BepiPred Server—positions aa 486–506 (153–173), E = part of a linear B cell epitope (• represents a residue predicted not to be part of an epitope) [85] (**d**).

Two important S1 epitopes were determined with Expasy Server—ProtScale [81,82] using the hydrophobicity scale of Kyte & Doolittle, with a sliding block of 11 amino acids (Figure 6a,b). The probable binding site in the region 153–173 was confirmed by the Informational Spectrum Method (ISM, Figure 6c) using electron-ion interaction pseudopotential (EIIP) [18,83,84]. The ISM located the bioactive *hot spot* at the exposed (E) position Q165 (Q498) within the region of S1 RBD fragment 153–173, which corresponds to the S1 amino acid motif 486–506, empirically shown to be important for the virus-receptor binding [66,68,69,73]. Identification of B cell epitopes by means of the Bepi Pred Server [85], presented in Figure 6d, confirmed the importance of this region and the result of ISM.

The docking of the 5′ → 3′ antisense peptide KKKKYRNIRRPG, 3′ → 5′ antisense peptide KLTIKGDVSIPKVGWLPQPIV, and peptide LVMGLNVWLRYSK to the RBD of S1 SARS-CoV-2 protein was done using the CABS-dock server (Figure 3, Figure 4 and Figure 5 and Table A2, Table A3 and Table A4) [28]. CABS-dock is a useful tool in the exploration of possible peptide binding sites [28,86]. It is a global docking procedure characterized by explicit, fully flexible docking simulation and clustering-based scoring [28,86,87]. The CABS model is based on a knowledge-based parameterization of the molecular interactions, which consist of several statistical potentials [28,86]. It describes the excluded volumes of the united atoms, a model of the main chain hydrogen bonds, and the side-chain contact potentials [86]. The results of the CABS-dock procedure accurately predicted the binding of both antisense peptides and peptide LVMGLNVWLRYSK to the S1 RBD receptor epitope FNCYFPLQSYGFQPTNGVGYQ (Figure 3, Figure 4 and Figure 5 and Table A2, Table A3 and Table A4).

### 4.2. Heuristic Antinsense Peptide Design (HAPD)

**Scheme 1:**
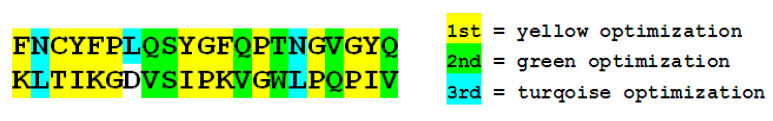
Heuristic method based on a three-step optimization procedure for APD.

A simple heuristic antisense peptide design (HAPD) was completed using the optimization procedure based on three steps for complementary, i.e., sense–antisense, pairs selection (Scheme 1). The steps were denoted by colors (e.g., yellow, green, and turquoise). The first step, yellow, was used to model the antisense peptide “consensus skeleton”, based on the selection of pairs specified by both complementary translation directions (3′ → 5′ and 5′ → 3′, Table 2). The first step reduced the number of random peptides in the HAPD model by the factor of 4.096 × 10^15^, i.e., from 20^12^ to 1. The second optimization step was green, and it specified the direction of translation for the “skeleton” (3′ → 5′ or 5′ → 3′). For the design of the antisense peptide KLTIKGDVSIPKVGWLPQPIV, we chose the 3′ → 5′ direction. The green criterion was the maximal hydropathy distance between polar–nonpolar amino acid clusters specified by the second base of the SGC table (A and U) and minimal hydropathy distance between neutral–neutral amino acid clusters specified by the second base of the SGC table (G and C) [7,8,17,19,24,88]. We applied the standard hydropathy distance criterion based on the Kyte and Doolittle scale [7,17,82]. For the clusters specified by the 3′ → 5′ complements of the modeled S1 sequence FNCYFPLQSYGFQPTNGVGYQ, the result is clear—QV, SS, and TW pairs were used (Table 2). The second (green) step enabled further reduction of elements by the factor of 6.4 × 10^7^, i.e., from 20^6^ to 1. In the third, turquoise, step there was only one possible antisense pairing left, containing the letters N, D, or E at the peptide aa position No. 7. This last step further reduced the number of random peptide pairs from 8000 (20^3^) to only 3. Three amino acids (N, D, and E) have an identical hydropathy value (−3.5) and hydropathy difference (Δ = 7.3) in relation to their only matching 3′ → 5′ complementary transcript L (3.8) [7,82]. Consequently, we randomly selected aspartic acid (D) for the final antisense sequence KLTIKGDVSIPKVGWLPQPIV. A similar strategy could also be applied to the 5′ → 3′ direction complements or to combinations of both procedures. For specific applications in immunochemistry and other disciplines, similar heuristic procedures for APD may also be combined with other heuristic bioinformatic methods such as, e.g., the basic local alignment search tool (BLAST) [18,20]. This procedure could also be used to model different natural or artificial (de novo) gene and protein constructs. 

A useful heuristic algorithm reduces the size of the solution space for a problem and also enriches the density of hits in the subset. Instead of 20^n^ randomly generated peptides for a sequence of length n within a full sample space, HAPD generates the average subset number of 1.35^n^ peptides in 3′ → 5′ and 2.6^n^ peptides in 5′ → 3′ translation direction. Consequently, the probability of deriving a pentapeptide binder using the random peptide design approach should be 3.125 × 10^−7^. In contrast to the random library approach, the average probabilities to detect pentapeptide binders using HAPD 3′ → 5′ and 5′ → 3′ methods would be 0.223 and 0.0084, respectively. Therefore, it is not surprising that the validity of HAPD was confirmed for more than 50 ligand-acceptor systems [1,2,3,4,5,6,7,8,9,10,11,12,13,14,15,16,17,18,19,20,21,22]. 

## 5. Conclusions

The applicability of APT was confirmed recently for the magnetic particle enzyme immunoassay (MPEIA, Figure 2b) and immunohistochemical procedures [19,20]. This opens a perspective for the development of a new class of efficient immunochemical assays based on short peptide technology [18,19,20]. Additionally, it was also shown that modern computational methods enable a new approach to the studies of sense and antisense peptide interactions [20]. Several free web-based services for protein structure prediction and modeling (e.g., I-TASSER, Phyre2, PEP-FOLD 3, CABS-dock) enable accurate protein-peptide docking, i.e., in silico search for the peptide binding sites [20,28,80,86,87,89,90,91].

Small molecules and peptides may be also used for blocking protein-protein and protein-peptide interactions. In addition to NMR and X-ray crystallographic methods and mutational data, computational and virtual spectroscopy methods—such as the informational spectrum method (ISM)—could be also used to define hot spots in proteins [18,83,84]. An APT-based approach is also useful for peptide interaction and pharmacophore modeling [32,35]. The application of artificial proteins in the context of APT is also a plausible method to derive new antisense modulators of the protein interactions [19,24,88,91,92].

APT could be easily adapted to magnetic and polystyrene bead assays, conventional ELISAs, and multiplex assays, so it is possible to achieve two major lines of quick and sensitive assay development: 1. MPEIAs read with appropriate absorbance readers, and 2. Multiplex ELISAs read with appropriate imagers (e.g., with a high-resolution chemiluminescence readers for printed microtiter plates) [19,20,93,94].

Developing new immunoassays is important for situations such as the SARS-CoV-2 infection outbreak (and COVID-19 disease) due to the possibility to design—in a relatively short time—quick, inexpensive, and simple assays that could be automated to obtain medium/high throughput screenings of particular binders, peptide motifs, and antibodies, etc. If carefully selected, such laboratory techniques enable the experimental application of different laboratory procedures which, depending on the experimental design, may be used for:

- selection of different targets and evaluation of complementary (sense–antisense) peptide binding;
- quantification of specific antibodies, peptides, and proteins;
- design of MPEIAs and Multiplex ELISAs tailored for a specific purpose.

The benefits of APT outweigh the costs of medium/high throughput screening and random peptide libraries and could lead to considerable savings in time and money. Practical applications and benefits of APT application are:

1. Quick design and validation of the complementary ligands and acceptors;
2. Computational validation and virtual screening of different protein and peptide structures;
3. Rationalization of peptide library screening;
4. The tests can be produced in a short period of time;
5. The tests will be made composite (according to the LEGO principle) and will consist of less expensive and commercially available components;
6. The time required to obtain results is shorter (since no antibody production is needed);
7. The test enables large quantity sample testing using standard laboratory equipment (since it does not require special reagents or complicated sampling processing);
8. The tests are likely to prove important for the investigation of the immune response, disease pathogenesis, and clinical outcome of different infections;
9. Designed antisense peptides (and anti-antisenses [21]) may also provide a basis for further development of vaccines and lead compounds for different diseases;
10. Detection of mutant strains is quicker since new antisense peptide motifs could be synthesized, evaluated for binding, and easily linked to magnetic particles in a short period of time, which avoids the antibody production process;
11. A green chemistry approach significantly reduces or avoids the loss of animal life.

## Figures and Tables

**Table 1:** Standard genetic code table.

First Letter (5′)	Second Letter				Third Letter (3′)
	U	C	A	G	
U	F	S	Y	C	U
	F	S	Y	C	C
	L	S	stop	stop	A
	L	S	stop	W	G
C	L	P	H	R	U
	L	P	H	R	C
	L	P	Q	R	A
	L	P	Q	R	G
A	I	T	N	S	U
	I	T	N	S	C
	I	T	K	R	A
	M	T	K	R	G
G	V	A	D	G	U
	V	A	D	G	C
	V	A	E	G	A
	V	A	E	G	G

**Table 2:** Direction of translation specifies amino acid pairing.

Amino Acid	Antisense 3′ → 5′	Antisense 5′ → 3′	Consensus
F	K	K, E	K
L	D, E, N	E, Q, K	E
I	Y	N, D, Y	Y
M	Y	H	
V	H, Q	H, D, N, Y	H
S	S, R	G, R, T, A	R
P	G	G, W, R	G
T	W, C	G, S, C, R	C
A	R	R, G, S, C	R
Y	M, I	I, V	I
H	V	V, M	V
Q	V	L	
N	L	I, V	
K	F	F, L	F
D	L	I, V	L
E	L	L, F	L
C	T	T, A	T
W	T	P	
R	A, S	A, S, P, T	A, S
G	P	P, S, T, A	P

**Table 3:** Correlations between amino acid factors and preference scales in frames 0 and +1 (−2).

Parameter	Polarity (20 aa)	Polarity (X_0_, 12 aa)	Diversity (X_1_, 11 aa)
GUA—nucleobase preference	−0.54 *	−0.63 *	0.71 *
PUR—nucleobase preference	−0.07	−0.49 *	0.82 *
PYR—nucleobase preference	0.06	0.49 *	–0.85 *

* *p* < 0.05 (Pearson’s *R*); aa = amino acid.

## Supplementary Materials

The following are available online at https://www.mdpi.com/article/10.3390/ijms22179106/s1, Table S1: b9526e5e0bfb562_model_1, Table S2: f667dc5b0700afa_model_1, Table S3: 70f677205828130_model_1, Table S4: model_S1RBD_aa334-523, Table S5: model_S1RBD_aa486-506, Table S6: model_ARBD.

## Appendix A

**Table A1:** Fragment of S1 RBD sequence aa 334–523 of SARS-CoV-2was used for CABS-dock analyses [28].

NLCPFGEVFNATRFASVYAWNRKRISNCVADYSVLYNSASFSTFKCYGVSPTKLNDLCFTNVYADSFVIRGDEVRQIAPGQTGKIADYNYKLPDDFTGCVIAWNSNNLDSKVGGNYNYLYRLFRKSNLKPFERDISTEIYQAGSTPCNGVEGFNCYFPLQSYGFQPTNGVGYQPYRVVVLSFELLHAPAT

**Table A2:** Pairs of peptide/receptor residuals closer than 4.5 Å in the selected complex using the CABS-dock method [28]. Peptide sequence was KKKKYRNIRRPG [75] and receptor region was aa 334–523 of RBD S1 SARS-CoV-2.

Receptor Residue	Peptide Residue	Receptor Residue	Peptide Residue	Receptor Residue	Peptide Residue
SER A 161	ASN B 7	TYR A 162	TYR B 5	TYR A 162	ARG B 6
GLN A 160	PRO B 11	GLN A160	GLY B 12	SER A 161	ARG B 6
GLN A 160	LYS B 4	GLN A 160	ARG B 6	GLN A 160	ILE B 8
PHE A 157	ARG B 10	PHE A 157	PRO B 11	LEU A 159	ILE B 8
TYR A 156	GLY B 12	PHE A 157	ILE B 8	PHE A 157	ARG B 9
GLU A 151	ARG B 9	TYR A156	ARG B 10	TYR A156	PRO B 11
LEU A 122	GLY B 12	PHE A 123	GLY B 12	TYR A 140	GLY B 12
TYR A 116	ASN B 7	TYR A 118	ARG B 6	TYR A 120	ARG B 6
LYS A 84	LYS B 2	LYS A 84	LYS B 4	ILE A 85	ARG B 6
GLN A 76	LYS B 3	THR A 82	LYS B 2	GLY A 83	LYS B 2
ARG A 75	LYS B 1	ARG A 75	LYS B 2	GLN A 76	LYS B 2
GLU A 73	LYS B 3	GLU A 73	LYS B 4	GLU A 73	ARG B 6
ARG A 70	TYR B 5	ASP A 72	LYS B 2	ASP A 72	LYS B 3
ILE A 69	ARG B 6	ARG A 70	LYS B 3	ARG A 70	LYS B 4

**Table A3:** Sense-antisense pairs of peptide/receptor residuals closer than 4.5 Å in the selected complex using CABS-dock method [28]. Antisense peptide sequence was KLTIKGDVSIPKVGWLPQPIV, and the receptor sense region was aa 334–523 of RBD S1 SARS-CoV-2.

Receptor Residue	Peptide Residue	Receptor Residue	Peptide Residue	Receptor Residue	Peptide Residue
TYR A 172	ASP B 7	TYR A 162	ASP B 7	GLY A 163	SER B 9
SER A 161	ILE B 10	SER A 161	VAL B 8	SER A 161	SER B 9
GLN A 160	TRP B 15	GLN A 160	VAL B 8	GLN A 160	ILE B 10
LEU A 159	VAL B 13	PHE A 157	GLY B 14	LEU A 159	ILE B 10
PHE A 157	VAL B 13	TYR A 156	LEU B 16	TYR A 156	GLN B 18
TYR A 156	TRP B 15	TYR A 156	VAL B 13	TYR A 156	GLY B 14
CYS A 155	LEU B 16	ASN A154	PRO B 19	CYS A 155	GLY B 14
GLU A 151	GLY B 14	GLU A 151	LYS B 12	GLU A 151	VAL B 13
GLYA 143	PRO B 19	ALA A 142	PRO B 19	ALA A 142	ILE B 20
ALA A 142	GLN B 18	TYR A 140	GLN B 18	TYR A 140	ILE B 20
ARG A 124	LEU B 2	PHE A 123	LEU B 2	ARG A 124	LYS B 1
LEU A 122	TRP B 15	LEU A 122	LEU B 2	LEU A 122	THR B 3
TYR A 120	VAL B 8	TYR A 120	LYS B 5	TYR A 120	ASP B 7
ILE A 85	LYS B 5	LYS A 84	LEU B 2	LYS A 84	THR B 3
LYS A 84	LYS B 1	GLU A 73	LYS B 5	GLN A 76	LYS B 5
ARG A 70	LYS B 5				

**Table A4:** Pairs of peptide/receptor residuals closer than 4.5 Å in the selected complex using CABS-dock method [28]. Peptide sequence was LVMGLNVWLRYSK [29] and receptor was region aa 334–523 of RBD S1 SARS-CoV-2.

Receptor Residue	Peptide Residue	Receptor Residue	Peptide Residue	Receptor Residue	Peptide Residue
TYR A 172	LYS B 13	GLY A 163	TYR B 11	TYR A 172	SER B 12
SER A 161	TYR B 11	SER A 161	LEU B 9	SER A 161	ARG B 10
SER A 161	TRP B 8	GLN A 160	SER B 12	SER A 161	VAL B 7
GLN A 160	ARG B 10	GLN A 160	VAL B 7	GLN A160	TRP B 8
LEU A 159	TRP B 8	LEU A 159	ASN B 6	LEU A 159	VAL B 7
LEU A 159	LEU B 5	PHE A 157	ASN B 6	PHE A 157	TRP B 8
TYR A 156	ARG B 10	CYS A 155	TRP B 8	TYR A 156	TRP B 8
GLU A 138	ASN B 6	THR A 137	LEU B 5	THR A 137	ASN B 6
THR A 137	GLY B 4	ILE A 135	VAL B 2	THR A 137	MET B 3
LEU A 119	VAL B 7	TYR A 116	VAL B 7	LEU A 119	VAL B 2
LYS A 84	LYS B 13	GLN A 76	LYS B 13	LYS A 84	SER B 12
GLU A 73	LYS B 13	ARG A 70	LYS B 13	ASP A 72	LYS B 13
ARG A 70	SER B 12	ARG A 70	ARG B 10	ARG A 70	TYR B 11
ALA A 19	VAL B 2	TYR A 18	VAL B 2	TYR A 18	MET B 3
ARG A 13	LEU B 1				

## References

[1] Root-Bernstein R.S. (1982). Amino acid pairing. J. Theor. Biol..

[2] Loo J.A., Holsworth D.D., Root-Bernstein R.S. (1994). Use of electrospray ionization mass spectrometry to probe antisense peptide interactions. Biol. Mass Spectrom..

[3] Holsworth D.D., Kiely J.S., Root-Bernstein R.S., Overhiser R.W. (1994). Antisense-designed peptides: A comparative study focusing on possible complements to angiotensin II. Pept. Res..

[4] Root-Bernstein R.S., Holsworth D.D. (1998). Antisense peptides: A critical mini-review. J. Theor. Biol..

[5] Root-Bernstein R.S. (2005). Peptide self-aggregation and peptide complementarity as bases for the evolution of peptide receptors: A review. J. Mol. Recognit..

[6] Root-Bernstein R. (2015). How to make a non-antigenic protein (auto) antigenic: Molecular complementarity alters antigen processing and activates adaptive-innate immunity synergy. Anticancer Agents Med. Chem..

[7] Blalock J.E. (1990). Complementarity of peptides specified by ‘sense’ and ‘antisense’ strands of DNA. Trends. Biotechnol..

[8] Blalock J.E. (1995). Genetic origin of protein shape and interaction rules. Nat. Med..

[9] Biro J.C. (2007). The proteomic code: A molecular recognition code for proteins. Theor. Biol. Med. Model..

[10] Mekler L.B. (1970). Specific selective interaction between amino acid residues of the polypeptide chains. Biophys. USSR.

[11] Mekler L.B., Idlis R.G. (1981). Construction of models of three-dimensional biological polypeptide and nucleoprotein molecules in agreement with a general code which determines specific linear recognition and binding of amino acid residues of polypeptides to each other and to the trinucleotides of polynucleotides. Depos. Doc. VINITI.

[12] Tropsha A., Kizert J.S., Chaiken I.M. (1992). Making sense from antisense: A review of experimental data and developing ideas on sense-antisense peptide recognition. J. Mol. Recognit..

[13] Siemion I.Z., Cebrat M., Kluczyk A. (2004). The problem of amino acid complementarity and antisense peptides. Curr. Protein Pept. Sci..

[14] Heal J.R., Roberts G.W., Raynes J.G., Bhakoo A., Miller A.D. (2002). Specific interactions between sense and complementary peptides: The basis for the proteomic code. ChemBioChem.

[15] Miller A.D. (2015). Sense-antisense (complementary) peptide interactions and the proteomic code; potential opportunities in biology and pharmaceutical science. Expert. Opin. Biol. Ther..

[16] Štambuk N. (1998). On the genetic origin of complementary protein coding. Croat. Chem. Acta.

[17] Štambuk N., Konjevoda P., Boban-Blagaić A., Pokrić B. (2005). Molecular recognition theory of the complementary (antisense) peptide interactions. Theory Biosci..

[18] Štambuk N., Manojlović Z., Turčić P., Martinić R., Konjevoda P., Weitner T., Wardega P., Gabričević M. (2014). A simple three-step method for design and affinity testing of new antisense peptides: An Example of Erythropoietin. Int. J. Mol. Sci..

[19] Štambuk N., Konjevoda P., Turčić P., Kövér K., Novak Kujundžić R., Manojlović Z., Gabričević M. (2018). Genetic coding algorithm for sense and antisense peptide interactions. Biosystems.

[20] Štambuk N., Konjevoda P., Turčić P., Šošić H., Aralica G., Babić D., Seiwerth S., Kaštelan Ž., Kujundžić R.N., Wardega P. (2019). Targeting Tumor Markers with Antisense Peptides: An Example of Human Prostate Specific Antigen. Int. J. Mol. Sci..

[21] McGuire K.L., Holmes D.S. (2005). Role of complementary proteins in autoimmunity: An old idea re-emerges with new twists. Trends Immunol..

[22] Dayhoff G.W., van Regenmortel M.H.V., Uversky V.N. (2020). Intrinsic disorder in protein sense-antisense recognition. J. Mol. Recognit..

[23] Štambuk N., Kopjar N., Šentija K., Garaj-Vrhovac V., Vikić-Topić D., Marušić-Della Marina B., Brinar V., Trbojević-Čepe M., Žarković N., Ćurković B. (1998). Cytogenetic effects of met-enkephalin (peptid-M) on human lymphocytes. Croat. Chem. Acta.

[24] Štambuk N., Konjevoda P. (2020). Determining amino acid scores of the genetic code table: Complementarity, structure, function and evolution. Biosystems.

[25] Van Regenmortel M.H.V. (2009). Synthetic peptide vaccines and the search for neutralization B cell epitopes. Open Vaccine J..

[26] Uversky V.N., van Regenmortel M.H.V. (2021). Mobility and disorder in antibody and antigen binding sites do not prevent immunochemical recognition. Crit. Rev. Biochem. Mol. Biol..

[27] Edmundson A.B., Ely K.R., Herron J.N., Cheson B.D. (1987). The binding of opioid peptides to the Mcg light chain dimer: Flexible keys and adjustable locks. Mol. Immunol..

[28] Ciemny M., Kurcinski M., Kamel K., Kolinski A., Alam N., Schueler-Furman O., Kmiecik S. (2018). Protein-peptide docking: Opportunities and challenges. Drug Discov. Today.

[29] Pomplun S., Jbara M., Quartararo A.J., Zhang G., Brown J.S., Lee Y.C., Ye X., Hanna S., Pentelute B.L. (2021). De novo discovery of high-affinity peptide binders for the SARS-CoV-2 spike protein. ACS Cent. Sci..

[30] Pomplun S. (2021). Targeting the SARS-CoV-2-spike protein: From antibodies to miniproteins and peptides. RSC Med. Chem..

[31] Bowen J., Schneible J., Bacon K., Labar C., Menegatti S., Rao B.M. (2021). Screening of yeast display libraries of enzymatically treated peptides to discover macrocyclic peptide ligands. Int. J. Mol. Sci..

[32] Turčić P., Štambuk N., Konjevoda P., Kelava T., Gabričević M., Stojković R., Aralica G. (2015). Modulation of γ_2_-MSH hepatoprotection by antisense peptides and melanocortin subtype 3 and 4 receptor antagonists. Med. Chem..

[33] Graham P. (2001). Instant Notes in Medicinal Chemistry.

[34] Woese C.R., Dugre D.H., Saxinger W.C., Dugre S.A. (1966). The molecular basis for the genetic code. Proc. Natl. Acad. Sci. USA.

[35] Houra K., Turčić P., Gabričević M., Weitner T., Konjevoda P., Štambuk N. (2011). Interaction of α-Melanocortin and Its Pentapeptide Antisense LVKAT: Effects on Hepatoprotection in Male CBA Mice. Molecules.

[36] Mohri M., Rezapoor H. (2009). Effects of heparin, citrate, and EDTA on plasma biochemistry of sheep: Comparison with serum. Res. Vet. Sci..

[37] Minarova H., Palikova M., Mares J., Syrova E., Blahova J., Faldyna M., Ondrackova P. (2019). Optimisation of the lymphocyte proliferation assay in rainbow trout (*Oncorhynchus mykiss*). Vet. Med..

[38] Root-Bernstein R. (2007). Simultaneous origin of homochirality, the genetic code and its directionality. Bioessays.

[39] Root-Bernstein R. (2010). Experimental test of L- and D-amino acid binding to L- and D-codons suggests that homochirality and codon directionality emerged with the genetic code. Symmetry.

[40] Turčić P., Bradamante M., Houra K., Štambuk N., Kelava T., Konjevoda P., Kazazić S., Vikić-Topić D., Pokrić B. (2009). Effects of α-Melanocortin Enantiomers on Acetaminophen-Induced Hepatotoxicity in CBA Mice. Molecules.

[41] Arquès D.G., Michel C.J. (1996). A complementary circular code in the protein coding genes. J. Theor. Biol..

[42] Michel C.J. (2015). The maximal C3 self-complementary trinucleotide circular code X in genes of bacteria, eukaryotes, plasmids and viruses. J. Theor. Biol..

[43] Bartonek L., Braun D., Zagrovic B. (2020). Frameshifting preserves key physicochemical properties of proteins. Proc. Natl. Acad. Sci. USA.

[44] Štambuk N. (1999). On circular coding properties of gene and protein sequences. Croat. Chem. Acta.

[45] Štambuk N. (2000). Universal metric properties of the genetic code. Croat. Chem. Acta.

[46] Wichmann S., Ardern Z. (2019). Optimality in the standard genetic code is robust with respect to comparison code sets. Biosystems.

[47] Wichmann S., Scherer S., Ardern Z. (2020). Computational design of genes encoding completely overlapping protein domains: Influence of genetic code and taxonomic rank. bioRxiv.

[48] Youvan D.C. Mathematics of the Genetic Code. https://www.youvan.com/MathematicsoftheGeneticCode-submit2-redacted.pdf.

[49] Füllen G., Youvan D.C. (1994). Genetic algorithms and recursive ensemble mutagenesis in protein engineering. Complex Int..

[50] Arkin A.P., Youvan D.C. (1992). An algorithm for protein engineering: Simulations of recursive ensemble mutagenesis. Proc. Natl. Acad. Sci. USA.

[51] Dila G., Michel C.J., Thompson J.D. (2020). Optimality of circular codes versus the genetic code after frameshift errors. Bio. Syst..

[52] May E., Vouk M., Rosnick D. (2004). An error-correcting code framework for genetic sequence analysis. J. Frankl. Inst..

[53] Thompson J.D., Ripp R., Mayer C., Poch O., Michel C.J. (2021). Potential role of the X circular code in the regulation of gene expression. Biosystems.

[54] Seligmann H., Pollock D.D. (2004). The ambush hypothesis: Hidden stop codons prevent off-frame gene reading. DNA Cell Biol..

[55] Blanchet S., Cornu D., Hatin I., Grosjean H., Bertin P., Namy O. (2018). Deciphering the reading of the genetic code by near-cognate tRNA. Proc. Natl. Acad. Sci. USA.

[56] Blanchet S., Cornu D., Argentini M., Namy O. (2014). New insights into the incorporation of natural suppressor tRNAs at stop codons in Saccharomyces cerevisiae. Nucleic Acids Res..

[57] Solis A.D. (2015). Amino acid alphabet reduction preserves fold information contained in contact interactions in proteins. Proteins.

[58] Atchley W.R., Zhao J., Fernandes A.D., Drüke T. (2005). Solving the protein sequence metric problem. Proc. Natl. Acad. Sci. USA.

[59] Polyansky A.A., Zagrovic B. (2013). Evidence of direct complementary interactions between messenger RNAs and their cognate proteins. Nucleic Acids Res..

[60] Koonin E.V., Novozhilov A.S. (2017). Origin and evolution of the universal genetic code. Annu. Rev. Genet..

[61] Choi J., O’Loughlin S., Atkins J.F., Puglisi1 J.D. (2020). The energy landscape of −1 ribosomal frameshifting. Sci. Adv..

[62] Rozov A., Westhof E., Yusupov M., Yusupova G. (2016). The ribosome prohibits the G U wobble geometry at the first position of the codon–anticodon helix. Nucleic Acids Res..

[63] Fang Y., Treffers E.E., Li Y., Tas A., Sun Z., van der Meer Y., de Ru A.H., van Veelen P.A., Atkins J.F., Snijder E.J. (2012). Efficient −2 frameshifting by mammalian ribosomes to synthesize an additional arterivirus protein. Proc. Natl. Acad. Sci. USA.

[64] Van Regenmortel M.H.V. (2016). Structure-based reverse vaccinology failed in the case of HIV because it disregarded accepted immunological theory. Int. J. Mol. Sci..

[65] Moxon R., Reche P.A., Rappuoli R. (2019). Editorial: Reverse Vaccinology. Front. Immunol..

[66] Lan J., Ge J., Yu J., Shan S., Zhou H., Fan S., Zhang Q., Shi X., Wang Q., Zhang L. (2020). Structure of the SARS-CoV-2 spike receptor-binding domain bound to the ACE2 receptor. Nature.

[67] Ni W., Yang X., Yang D., Bao J., Li R., Xiao Y., Hou C., Wang H., Liu J., Yang D. (2020). Role of angiotensin-converting enzyme 2 (ACE2) in COVID-19. Crit. Care.

[68] Walls A.C., Park Y.-J., Tortorici M.A., Wall A., McGuire A.T., Veesler D. (2020). Structure, function, and antigenicity of the SARS-CoV-2 spike glycoprotein. Cell.

[69] Tai W., He L., Zhang X., Pu J., Voronin D., Jiang S., Zhou Y., Du L. (2020). Characterization of the receptor-binding domain (RBD) of 2019 novel coronavirus: Implication for development of RBD protein as a viral attachment inhibitor and vaccine. Cell. Mol. Immunol..

[70] Whisenant J., Burgess K. (2020). Blocking Coronavirus 19 Infection via the SARS-CoV-2 spike protein: Initial steps. ACS Med. Chem. Lett..

[71] Zhang G., Pomplun S., Loftis A.R., Loas A., Pentelute B.L. (2020). The first-in-class peptide binder to the SARS-CoV-2 spike protein. bioRxiv.

[72] Zhang G., Pomplun S., Loftis A.R., Tan X., Loas A., Pentelute B.L. (2020). Investigation of ACE2 N-terminal fragments binding to SARS-CoV-2 Spike RBD. bioRxiv.

[73] Glasgow A., Glasgow J., Limonta D., Solomon P., Lui I., Zhang Y., Nix M.A., Rettko N.J., Lim S.A., Zha S. (2020). Engineered ACE2 receptor traps potently neutralize SARS-CoV-2. Proc. Natl. Acad. Sci. USA.

[74] Elton T.S., Dion L.D., Bost K.L., Oparil S., Blalock J.E. (1988). Purification of an angiotensin II binding protein by using antibodies to a peptide encoded by angiotensin II complementary RNA. Proc. Natl. Acad. Sci. USA.

[75] Huang Y., Zhao R., Luo J., Xiong S., Shangguan D., Zhang H., Liu G., Chen Y. (2008). Design, synthesis and screening of antisense peptide based combinatorial peptide libraries towards an aromatic region of SARS-CoV. J. Mol. Recognit..

[76] Myers D.G. (2010). Social Psychology.

[77] Michalewicz Z., David B., Fogel D.B. (2004). How to Solve It: Modern Heuristics.

[78] Heuristic Algorithms. https://optimization.mccormick.northwestern.edu/index.php/Heuristic_algorithms.

[79] Young D.C. (2009). Computational Drug Design: A Guide for Computational and Medicinal Chemists.

[80] Yang J., Yan R., Roy A., Xu D., Poisson J., Zhang Y. (2015). The I-TASSER Suite: Protein structure and function prediction. Nat. Methods.

[81] Gasteiger E., Hoogland C., Gattiker A., Duvaud S., Wilkins M.R., Appel R.D., Bairoch A., Walker J.M. (2005). Protein Identification and Analysis Tools on the ExPASy Server. The Proteomics Protocols Handbook.

[82] Kyte J., Doolittle R.F. (1982). A simple method for displaying the hydropathic character of a protein. J. Mol. Biol..

[83] Veljkovic V., Veljkovic N., Esté J.A., Hüther A., Dietrich U. (2007). Application of the EIIP/ISM bioinformatics concept in development of new drugs. Curr. Med. Chem..

[84] Veljkovic V., Perovic V., Paessler S. (2020). Prediction of the effectiveness of COVID-19 vaccine candidates. F1000Research.

[85] Larsen J.E., Lund O., Nielsen M. (2006). Improved method for predicting linear B-cell epitopes. Immunome Res..

[86] Kurcinski M., Badaczewska-Dawid A., Kolinski M., Kolinski A., Kmiecik S. (2020). Flexible docking of peptides to proteins using CABS-dock. Protein Sci..

[87] Kurcinski M., Ciemny M.P., Oleniecki T., Kuriata A., Badaczewska-Dawid A.E., Kolinski A., Kmiecik S. (2019). CABS-dock standalone: A toolbox for flexible protein-peptide docking. Bioinformatics.

[88] Štambuk N., Konjevoda P. (2020). The temperature dependence of amino acid hydrophobicity data is related to the genetic coding algorithm for complementary (sense and antisense) peptide interactions. Data Brief..

[89] Kelley L.A., Mezulis S., Yates C.M., Wass M.N., Sternberg M.J. (2015). The Phyre2 web portal for protein modeling, prediction and analysis. Nat. Protoc..

[90] Derreumaux P., Tufféry P. (2012). PEP-FOLD: An updated de novo structure prediction server for both linear and disulfide bonded cyclic peptides. Nucleic Acids Res..

[91] Štambuk N., Konjevoda P. (2017). Structural and functional modeling of artificial bioactive proteins. Information.

[92] Štambuk N., Konjevoda P. (2017). The hydrophobic moment: An early bioinformatics method and de novo protein design. Science.

[93] Liu R., Liu J., Xie L., Wang M., Luo J., Cai X. (2010). A fast and sensitive enzyme immunoassay for brain natriuretic peptide based on micro-magnetic probes strategy. Talanta.

[94] Pickering J.W., Hoopes J.D., Groll M.C., Romero H.K., Wall D., Sant H., Astill M.E., Hill H.R. (2007). A 22-plex chemiluminescent microarray for pneumococcal antibodies. Am. J. Clin. Pathol..

